# Development of novel parameters for characterising scale morphology of wool fibre and its correlation with dye diffusion coefficient of acid dye

**DOI:** 10.1038/s41598-023-45689-w

**Published:** 2023-10-27

**Authors:** Subhadeep Paul, Andrew Hewitt, Sohel Rana, Parikshit Goswami

**Affiliations:** 1 Technical Textiles Research Centre, School of Arts and Humanities, University of Huddersfield, Queensgate, Huddersfield, HD1 3DH UK; 2 Department of Textile & Fibre Engineering, Indian Institute of Technology Delhi, Hauz Khas, New Delhi, 110016 India

**Keywords:** Chemistry, Materials science

## Abstract

This paper reports the development of novel surface parameters which can be used to characterise the scale structure of wool fibres obtained from different breeds. Scanning electron microscopy and subsequent image analysis technique were used to study wool fibres from Leicester, Dartmoor, Ryeland and Herdwick breeds of sheep. Novel scale parameters related to wool fibre’s effective chemical diffusion pathway were developed. Namely, the total scale perimeter per 100 µm fibre length and scale perimeter index, which is the total scale perimeter per 100 µm length divided by the fibre diameter. Wool fibres obtained from different breeds showed significant differences in their scale pattern with the change in fibre diameter. The scale perimeter per 100 µm length increased with the fibre diameter and showed a polynomial correlation. It was also demonstrated that an increase in the diameter of the wool fibre resulted in an increase in the apparent dye diffusion coefficient, which contrasts the established theory that finer fibres are associated with a higher dyeing rate. The increase in effective diffusion pathway (total scale perimeter per 100 µm) for the wool fibres (among different breeds) resulted in a higher dye diffusion rate at the initial phase of dyeing (liquor to surface).

## Introduction

Wool is a multi-functional natural fibre which is valued in the twenty-first century fibre market due to its unique properties such as high elongation, thermal insulation, capacity to absorb and hold moisture and high ignition temperature^1–3^. Wool products are used in diverse applications, not only in garments but also in other technical application areas including filtration, composites, furnishings and construction engineering^4–10^. The unique properties of wool fibres originate from the macroscopic and microscopic features of their structure.

Wool fibre typically contains two main types of morphological components, the outer cuticle (the scales) and the inner cortex, which makes up almost 90% of the fibre^11^. The cuticle layer has a high cystine content, making it chemically resistant (highly crosslinked structure) and is further subdivided into epicuticle, exocuticle and endocuticle. The outmost epicuticle consists of a hydrophobic F-layer made of polar and nonpolar long chain fatty acids^12, 13^.

Wool fibres from different sheep breeds exhibit significant difference in properties (diameter, length, crimp, chemical composition, etc.) and in surface morphology (scale patterns and dimensions)^14–16^. The diffusion of water molecules into the fibre core is strongly dependent on the intercellular gaps present between the scales^17, 18^. Similarly, other chemicals can also follow a similar path of diffusion assuming that the molecules are small enough to diffuse through the intercellular gaps. It has been hypothesised that these intercellular gaps can control both the amount of diffused chemicals and the rate of diffusion and therefore, are expected to have strong influence on the fibre properties^19^.

**Figure 1:**
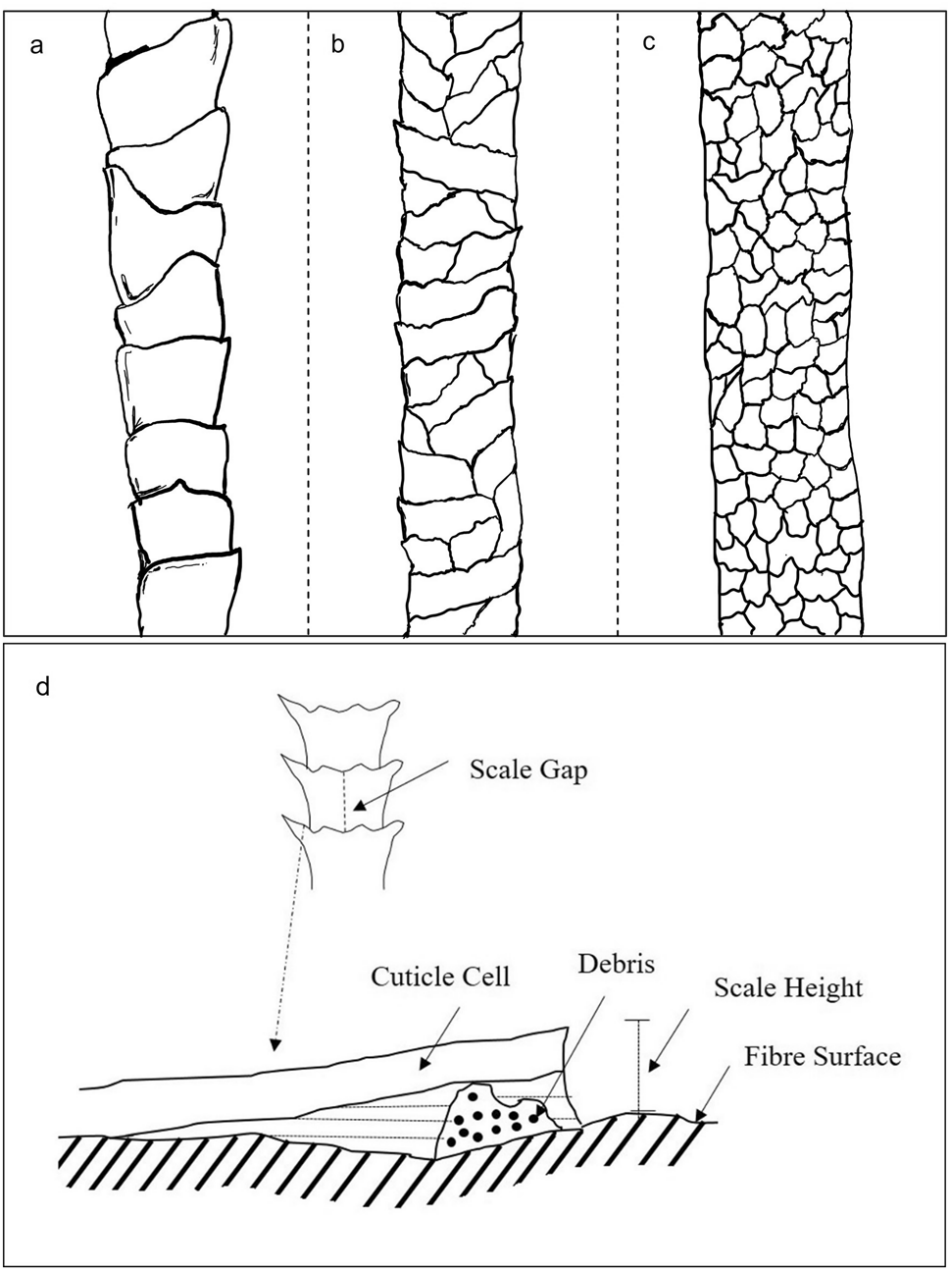
Different types of wool fibre scale pattern: (**a**) coronal, (**b**) coronal-reticulate and (**c**) reticulate and (**d**) schematic diagram representing the scale gap and scale height of wool fibres.

A lot of research has been carried out towards understanding the surface chemistry of wool fibres, revealing the presence of different types of amino acids and the distribution of fatty acids in epicuticle^20^. Image analysis of different animal fibres has also been performed using various methodologies^21^ to study their scale structures. For example, Blyth categorised wool scales into two types—coronal and reticulate forms^22^. The edges of the scales in the coronal pattern face away from the fibre axis and tend to grow around the fibre in a semi-circle. Wool fibres with a diameter of less than 25 µm show coronal pattern of scales^23^. Reticulate type scales are irregular in size and shape and appear randomly distributed on the surface of the fibre. Later studies discovered the coronal-reticulate scale pattern which is observed in the form of a network and divided into bands around the fibres running diagonally on the surface (Fig. 1a–c)^24^.

Wool fibre surface studies rely on the fibre microscopy. Methods of sample preparation have traditionally included immersing the fibre in a liquid medium or resin, mounting the fibre onto a medium, and building a fibre cast in a quick drying resin^21^ or plastic material^15^. These methods only reveal one side of the fibre, and an alternative method involves rolling the fibres between glass slides, one of which is coated with a synthetic resin that dries out quickly to solidify the mounted pattern^25^.

Fibre staining has also been used as an alternative approach to study the scales. Fibres are frequently pre-bleached and the scale dimensions are modified either by swelling of fibres or inserting debris (in the form of chemicals) between the cuticle and fibre surface, as shown in Fig. 1d^26^. All these methods rely on the penetration of chemicals through intercellular gaps which potentially could influence the structure through swelling or displacement^27, 28^.

Several studies have been reported in the literature to characterise different parameters related to the scale structure of wool fibres. Hausman et al. analysed several wool fibres obtained from different parts of the world (India, China, Turkey, Austria and Russia) to determine the average scale gap between two scales and reported that the number of scales increased with the increase in the diameter of the fibre^23, 29^. Robson et al. included more parameters related to the scales (such as circularity, rectangularity, aspect ratio, and scale interval) in their study that was carried out using the fibre casting method and an image analysis algorithm. There are, however, not much literature on how the scale gap could influence the diffusion of chemicals into the core of wool fibre, or could be related to different fibre properties^24, 30^.

The intercellular gaps present between the scales are considered to be the main diffusion pathway for chemical reagents (e.g. dyes) to penetrate into the fibre structure. This is because the rest of the outer cuticle is hydrophobic and relatively non-reactive towards many chemical reagents due to its highly crosslinked structure. Hence, the intercellular gaps influence the diffusion coefficient of these chemicals^31^ including dye molecules^32^. The first path of entrance of dye molecules into the fibre structure has also been confirmed by fluorescence microscopy as the intercellular gaps^33^. The total scale perimeter per 100 µm, used in this work signifies the total length of the cracks or opening present on the surface of a wool fibre. It can be hypothesised that higher the value of this parameter, more will be the intercellular gaps and chances for the dye molecules to penetrate inside the fibre, resulting in a higher diffusion coefficient. Diffusion coefficient (D) is defined as the change in mass of a substance that diffuse through a unit surface per unit concentration gradient. Such understanding about the diffusion coefficient value of different chemical reagents associated with wool fibre can be helpful for understanding the chemical sorption property of wool fibre and can be further utilised to manufacture filtration fabrics or materials which show high absorption of certain chemicals. The diffusion of dyes into fibre has been explained with the help of Fick’s law of diffusion^34^. But wool fibre in its diffusion curve follows a non-fickian diffusion at its very early stage of dyeing due to presence of surface barrier effect^32^. The calculation of D is a direct representation of rate of dyeing (kinetics). Diffusion in wool fibre occurs in three stages (i) from solution to the surface (ii) adsorption on surface and (iii) diffusion of dyes from surface to the core of the fibre^34^.

The mathematical equations for diffusion were developed based on the fact that it is constant (for the initial phase of dyeing), or it changes with the concentration of dye (which involves all the phases of diffusion). Diffusion coefficient of dyes has been reported to be dependent on factors like shape of the fibre, concentration gradient, time, temperature, rate of agitation and also, on the structure of the dye molecules^13, 35, 36^. Mainly Hill’s equation was used for the calculation of the diffusion coefficient, with time this equation has been modified by several researchers to make the models more accurate^37–40^. The Hills equation for the current research work represents an apparent diffusion coefficient which is assumed to stay constant for the initial phase of dyeing (i) Ref.^41^. There are multiple factors that affect the dye diffusion coefficient among them hydrodynamic boundary layer can have a significant impact on the dyeing kinetics^34^. As the dye molecules approach the fibre there is a ≈ 99% decrease in the velocity of the dye before it reaches the surface. At this point the diffusion of the dye molecules becomes an influencing factor. The distance at which this change in velocity is observed is called the hydrodynamic boundary layer. This layer can be effected by the circulation velocity of the liquor and the shape of the fibre^42^.

Most of the models consider wool fibre to be an infinite cylinder with no edges, but in a practical scenario wool fibre diameter varies within a single fibre and also the scales change their pattern from fibre to fibre. This may affect the hydrodynamic boundary layer thus influencing the diffusion coefficient. Theoretical modelling has always been modified from time to time to make the models more aligned with the experimental findings of dyeing. However, for natural fibre like wool it has always been very difficult to obtain an exact value for the diffusion coefficient and to conclude which theory is more suitable^37^. The knowledge of scale perimeter and SPI and its inclusion in the mathematical models of diffusion might make them more representative of real dyeing conditions.

Till date, research studies were mainly focused on the scale height which is the distance between the projected-out scale and the edge of the scale (see Fig. 1d). Technologies such as AFM (Atomic Force Microscopy) were used to obtain a more appropriate and accurate measurement of scale height of wool fibres^43, 44^. Scale height was observed to increase by 21%, on an average, in wet conditions as compared to dry conditions and the scale dimensions measured in dry conditions were more reproducible^43^. In wet conditions, there was swelling of wool fibre which was associated with the swelling of the cuticle, causing a change in the dimension of the scales. Therefore, the scale parameters measured using most of the previous methods were strongly dependant on the testing conditions (e.g. temperature, resin, pre-treatment of fibre, etc.). Moreover, the studied scale parameters have been mostly used for the identification of wool fibres and differentiate them from other hair fibres, but not for investigating the influence of surface morphology on the fibre functionality or properties. To bridge that gap in the existing literature, the present research reports novel scale parameters related to the intercellular gaps of wool fibre and discusses how these parameters can be correlated with fibre diameter as well as dye diffusion coefficient and fibre functionalities.

## Materials and methodology

### Materials

Wool fibres from four different UK sheep breeds were provided by Fleet Green Farm in the form of fleece. The breeds were Bluefaced Leicester (Leicester), Greyface Dartmoor (Dartmoor), Ryeland and Herdwick. Wool fibres were taken from the fleece randomly to avoid preferential sampling. Also, Welsh mountain wool fibre was obtained from British Wool and characterised to verify the experimental correlations achieved using the above four breeds of wool fibre.

Before analysis, wool fibres were scoured using sodium carbonate (Na_2_CO_3_) supplied by Sigma Aldrich, and ULTRAVON JUN (a nonionic detergent) manufactured by Huntsman Textile Effects (Germany) GmbH and supplied by Town End (Leeds). For dyeing of wool fibres, CI Acid Violet 90, sulphuric acid and sodium sulphate (Na_2_SO_4_) were used (supplied by Sigma Aldrich).

### Methodology

#### Scouring

20 g samples of wool were scoured using a four-bath process. The scouring recipe is listed in Table 1, which is based on the recipe used by Kadam et al.^45^. The scouring process was carried out using a liquor ratio of 1:50 with constant manual agitation. Prior to transfer between baths, the samples were squeezed to reduce transfer of liquor. After scouring, the samples were rinsed twice in an excess of cold water and squeezed before drying at 90 °C for 1 h in a convection oven. The samples were then conditioned at 20 ± 2 °C and 65 ± 3% RH for at least 24 h before analysis.

**Table 1:** Recipe for scouring of wool fibres.

Recipe	1st Bath	2nd Bath	3rd Bath	4th Bath
Na_2_CO_3_	1.33 g l^–1^	0.65 g l^–1^	0.65 g l^–1^	–
ULTRAVON JUN	7.5 g l^–1^	5.0 g l^–1^	2.5 g l^–1^	–
Temperature	60 °C	55 °C	50 °C	45 °C
Time	3 min	3 min	3 min	3 min

#### Carbonising of wool fibre

The scoured wool samples were carbonised based on a recipe reported by Park^46^, using sulphuric acid (70.0 g l^–1^) and ULTRAVON JUN (2.0 g l^–1^) in a liquor ratio of 1:20. The pH of the solution was 1.2. The wool fibres were immersed in the solution for 2 h at 20 ± 2 °C. The fibres were then removed, rinsed in cold water, dried and baked at 100 °C for 10 min. This was done so that all the vegetable matter present in wool could be removed as it becomes brittle. The fibres were manually crushed between two rollers so that the impurities were crushed and manually shaken to remove them. The wool fibres were rinsed once in an excess of cold water and then washed in a solution (1:20 liquor ratio) of sodium carbonate (53.0 g l^–1^) at room temperature. They were rinsed once again in an excess of cold water, squeezed and then dried overnight at room temperature. Damage to the F-layer of the wool fibres were evaluated by dyeing the fibres with Acid Red 1 at 2% omf, before and after carbonising. The wool fibres did not show any observable difference, hence no potential damage to the F-layer was confirmed.

#### Dyeing procedure

Pre-treated wool fibre samples from Leicester, Ryeland and Dartmoor (1 g) were dyed using a CI Acid Violet 90. The dyeing was carried out using laboratory-based Roaches Pyrotec IR dyeing machine using 2% CI Acid Violet 90 dye (on mass of fibre), 1% H_2_SO_4_ and 5% Glauber’s salt. The pH of the bath was maintained at 2.7 ± 0.2, dyeing temperature was kept 90 °C and the MLR (Material to Liquor ratio) was maintained at 1:50. The dyeing time was varied from 5 to 240 min.

#### Dyeing kinetics and diffusion coefficient calculation

The amount of dye adsorbed M_t_ (mg g^–1^) at time t was determined by the equation below.

1 $${M}_{t}=\frac{\left(Co-Ce\right)\times V}{W}$$

where C_o_ is the initial dye concentration (mg ml^–1^) in the solution, C_e_ is the left-over dye concentration in the solution after dyeing (mg ml^–1^), V is the initial volume of the solution (ml) and W is the weight of wool fibre (g).

The dye diffusion coefficient was calculated using the modified version of Hill’s equation for a finite bath^47^.

2 $$\frac{{M}_{t}}{{M}_{e}}=4{\left(\frac{Dt}{{\pi r}^{2}}\right)}^{1/2}$$

where M_e_ is the amount of dye adsorbed (mg g^–1^) at equilibrium, r is the radius of the fibre and D is the apparent diffusion coefficient. According to Eq. (2), the D can be calculated from the slope of the M_t_/M_e_ vs t^1/2^ curve. In this case the dye diffusion coefficient was considered to be constant in the initial phase of the dyeing and the modified equation of Hill (2) is valid only for a finite bath. The average of two sets of repeat for dyeing kinetics study was performed and the standard error of the calculated diffusion coefficient is reported in Table 3.

## Analysis

### Scanning electron microscopy (SEM)

SEM images were taken using Quanta FEG 250 scanning electron microscope (FEI Instruments) at different magnification levels ranging from 100 to 1000x. The samples were sputter coated with gold before measurement. The accelerating voltage was kept between 5 and 10 kV. The SEM images were used to evaluate different scale patterns, number of scales and the distribution of fibre diameter. For the measurement of diameter, a mean value of 50 readings for each breed was calculated and reported. Measurement of diameter for thousands of fibre via SEM images is a very tedious approach. For the mean fibre diameter, it will be beneficial to opt for an optical fibre diameter testing to have a better distribution and representation of the diameter data. The purpose of this research work was to test the initial feasibility of how scale structures of wool fibre could affect dyeing kinetics.

The scale analysis methodology employed in this work possesses some key advantages to preserve the structural properties of the fibres. The fibres were not immersed in a resin or any solvent which could influence the scale arrangement. The fibres did not undergo any kind of extension (to remove the crimp). Also, the measurement was performed in a dry condition. The fibres were scoured to remove contaminants such as dirt and wool grease so that the scales were perfectly visible under the microscope, but no additional pre-treatments (e.g. bleaching) were performed which could influence the scale arrangement.

Although in this method wool fibre samples were sputter coated with gold and measured in a vacuum, no previous literature indicated that this coating might change fibre and/or scale arrangement, and any changes that may occur are likely to be minimal as compared to immersion in a liquid. However, like most image analysis techniques reported earlier, this methodology characterised only one side of each fibre (a 2D image) rather than the entire 3D surface advanced technologies such as 3D SEM can be combined with the proposed method in future work for a more accurate measurement of scale morphology.

### Measurement of scale perimeter

**Figure 2:**
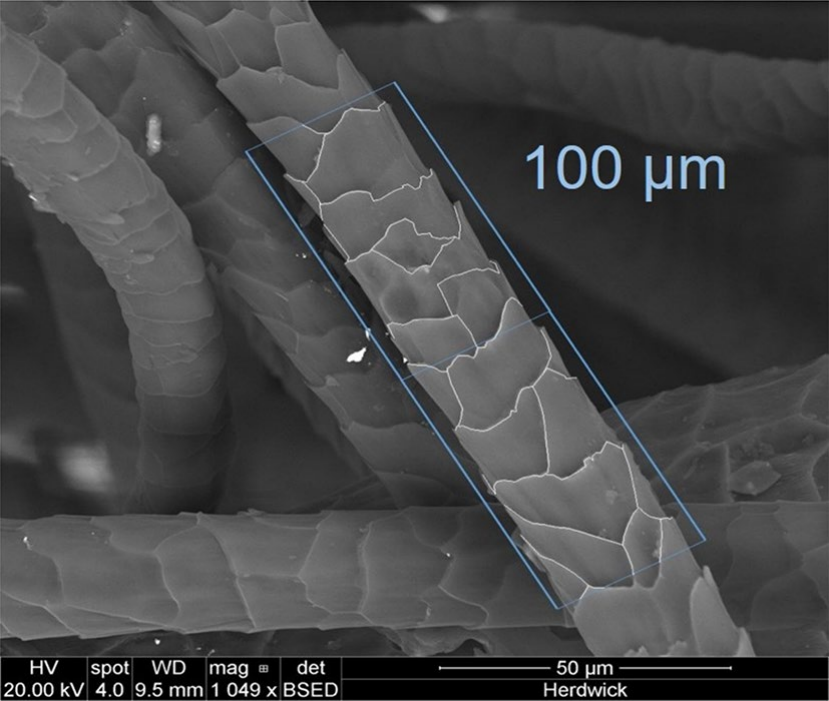
SEM image used for the calculation of scale perimeter per 100 µm.

The measurement of scale perimeter was performed from the SEM images using ImageJ software. Straight fibres were selected for measurements to eliminate errors caused by bending of fibres, as shown in Fig. 2. The number of visible scales were counted on the basis that a fully visible scale was counted as one and a partially visible scale was counted as 0.5. Perimeter and scale readings were taken per 100 µm length of the fibre. A parallel line was drawn along the fibre axis and 100 µm grid lines were drawn along the side of the fibre for measurement (Fig. 2). The measurement methodology has been simplified so that this methodology could be easily adopted.

Further, another scale parameter, namely scale perimeter index (SPI) was also calculated. SPI is a dimensionless parameter which is defined here as the total perimeter of all the visible scales on a 100 µm long 2D image of a single fibre divided by the diameter of that fibre. Therefore, SPI is defined as:

3 $$SPI=\frac{Total \,\,Scale \,\,Perimeter \,\,(per \,\,100 \,\mu m\,\, length \,\,of \,\,fibre)}{Fibre \,\,Diameter \,\,(\mu m)}$$

### UV–visible spectrophotometer

The dyeing solutions collected before and after dyeing were cooled to room temperature and their absorbance were measured by UV-visible spectrophotometer (Jasco V-730). The absorbance of the residual dye solution was calculated at 535 nm (the wavelength of maximum intensity, λ_max_) as deduced from the calibration curve of the dye (Fig. S2).

## Results and discussion

### Fibre diameter and its distribution

Wool fibres obtained from different breeds show variation in diameter between fibres, between fleece and also within breeds^48^. The mean fibre diameter data obtained from the SEM images of Leicester, Dartmoor, Ryeland and Herdwick wool fibres are listed in Table 2.

**Table 2:** Measured mean diameter for wool fibres from the four studied breeds.

Breed	Mean fibre diameter (µm)	CV (%)
Leicester	22	11.7
Ryeland	31	12.7
Herdwick	66	54.0
Dartmoor	72	22.5

**Figure 3:**
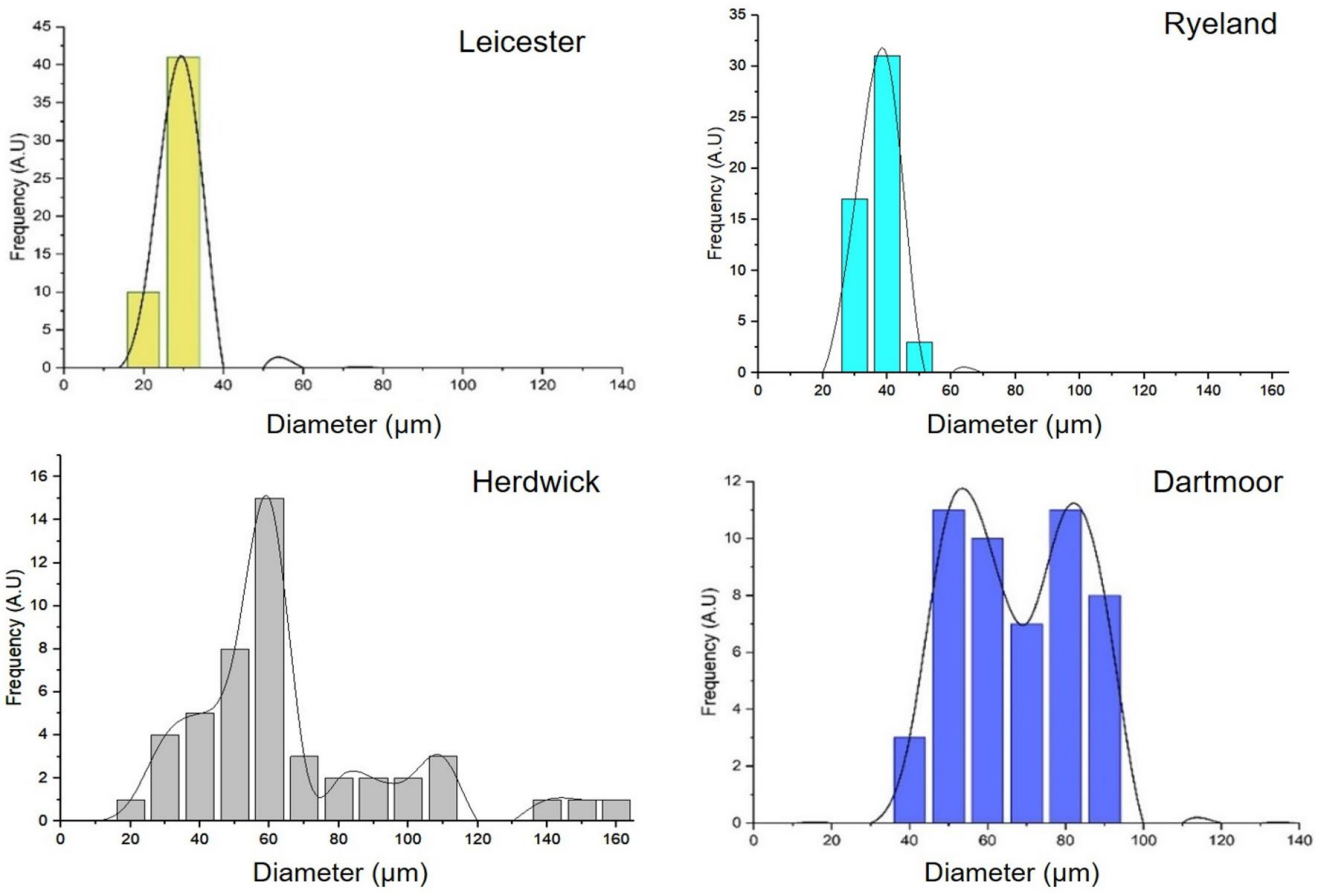
Frequency distribution of diameter for the studied wool fibre types.

The average diameter range of the studied UK wool fibres was very similar to the ones reported by Robson et al.^49^. Leicester wool fibres had a mean diameter of 22 µm and was the finest of the four varieties. The diameter distribution of Leicester fibre was narrower than the other wool types (Fig. 3). Dartmoor wool fibres had a mean fibre diameter of 72 µm which was, therefore, a coarser category, also commonly referred to as strong wool. The fibre diameter distribution of Dartmoor wool was narrower as compared to Herdwick and the range was between 43 and 100 µm. Herdwick and Ryeland wools had mean fibre diameter values of 66 and 31 µm, respectively. The diameter distributions of Herdwick and Dartmoor were significantly wider ranging from 26 to 162 µm and 43 to 100 µm, respectively (Fig. 3). The observed variation in wool fibre diameter was high because the wool fleeces were directly obtained from the farm where the fibres were not separated and graded according to the coarseness of fibres. Also testing more number of samples might transform the distribution curve of Herdwick and Dartmoor to a bell shaped distribution. The diameter distribution of Herdwick shows some of the fibres have diameter above 100 µm range and thus can be associated with kemp fibres.

### Scale pattern of wool fibres

**Figure 4:**
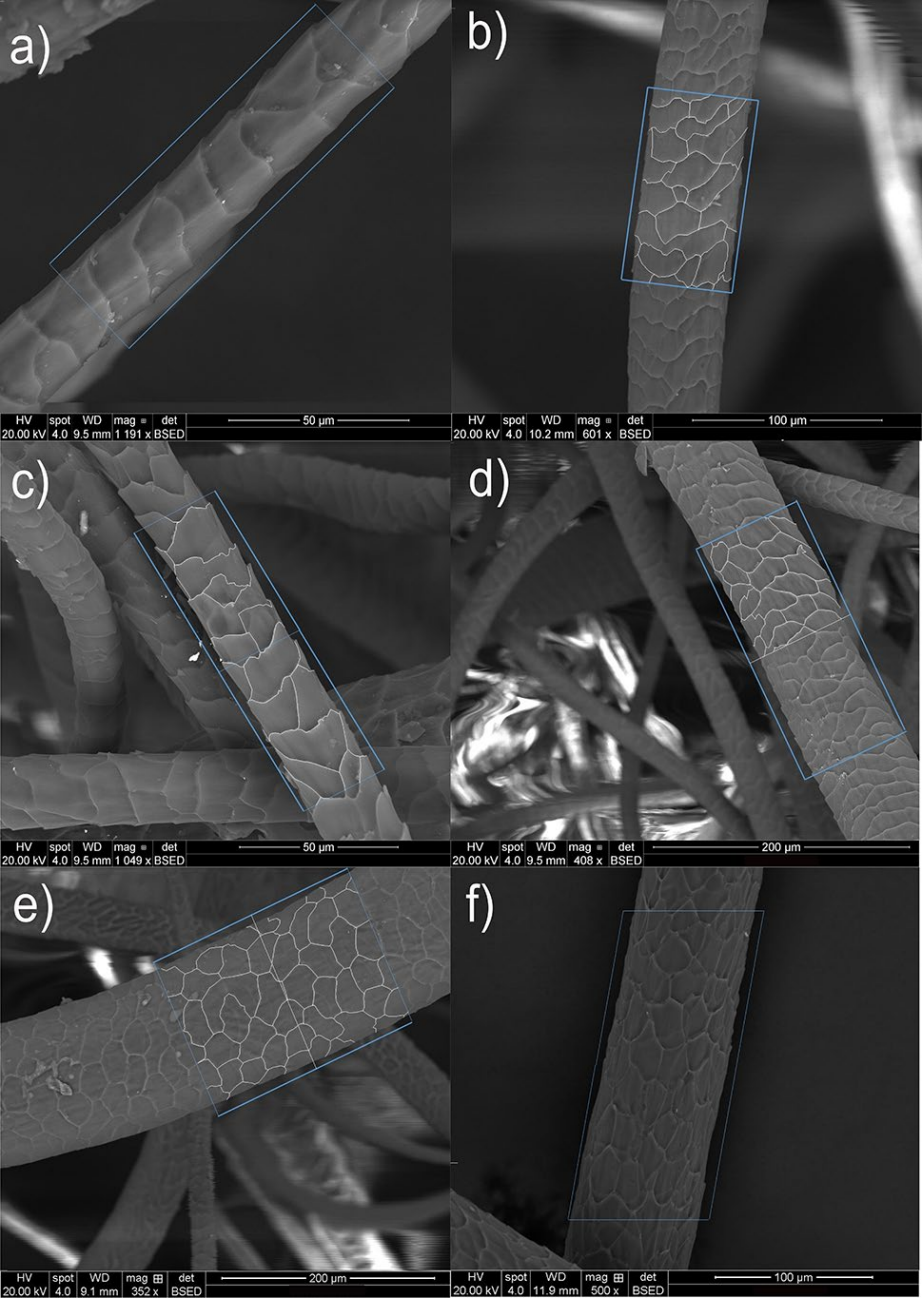
SEM images of visible scale pattern of (**a**) Leicester, (**b**) Ryeland, (**c**–**e**) Herdwick, and (**f**) Dartmoor fibres and their scale perimeter markings.

Leicester fibre exhibited a coronal pattern where each scale end formed a circular pattern on the fibre, whereas Dartmoor fibre showed a reticulate pattern in which scales were arranged randomly, as presented in Fig. 4. Herdwick fibre exhibited a wide range of fibre diameters and consequently, showed all three types of patterns: coronal (for fibre diameter < 25 µm), coronal-reticulate (for 25–50 µm fibre diameter) and reticulate (for fibre diameter > 50 µm), whereas only the former two patterns were observed in case of Ryeland wool. Ryeland fibres predominantly showed the coronal-reticulate pattern (Fig. 4).

The observations confirmed that the changes in the scale pattern of wool fibres are related to the fibre diameter. The number of scales and the scale pattern in Merino wool^14^ are different from the pattern shown by the coarser Dartmoor wool. The number of scales in Merino wool fibres are fewer and the pattern is the coronal type, which is similar to Leicester wool, the finest of the four varieties studied in this research^50^. Dartmoor (mean diameter 72 µm) wool showed a higher number of scales than Leicester (mean diameter 22 µm) due to the finer diameter of Leicester wool. Thus, the number of scales in the studied wool fibres per 100 µm increased with the fibre diameter. This can be clearly observed from the correlation curve plotted between the number scales and fibre diameter, as discussed in section “Total scale perimeter and scale perimeter index (SPI)”.

### Total scale perimeter and scale perimeter index (SPI)

It is hypothesised that the number of active molecules entering into the core of the fibre depends on the dimension of the diffusion pathway between the scales. As the number of gaps increases for a specific length of fibre (e.g. 100 µm), the amount of chemical uptake by the fibre should also increase. This is in agreement with the earlier observation that the generation of cracks on the surface of wool fibres improved the colour yield by allowing more spaces for the dye molecules to enter^51^. As per the discussion in Section “Scale pattern of wool fibres”, different types of wool fibre showed different scale patterns and therefore, it can be hypothesised that their effective diffusion pathway could also be significantly different^52^. This in turn may result in different chemical absorption behaviours for different breeds of wool fibre. To investigate this further and to use effective diffusion pathway for categorising wool fibres according to their chemical adsorption property, a new scale parameter, called total scale perimeter per 100 µm, was calculated as a measure of the total diffusion paths present on the surface within a fibre length of 100 µm.

**Figure 5:**
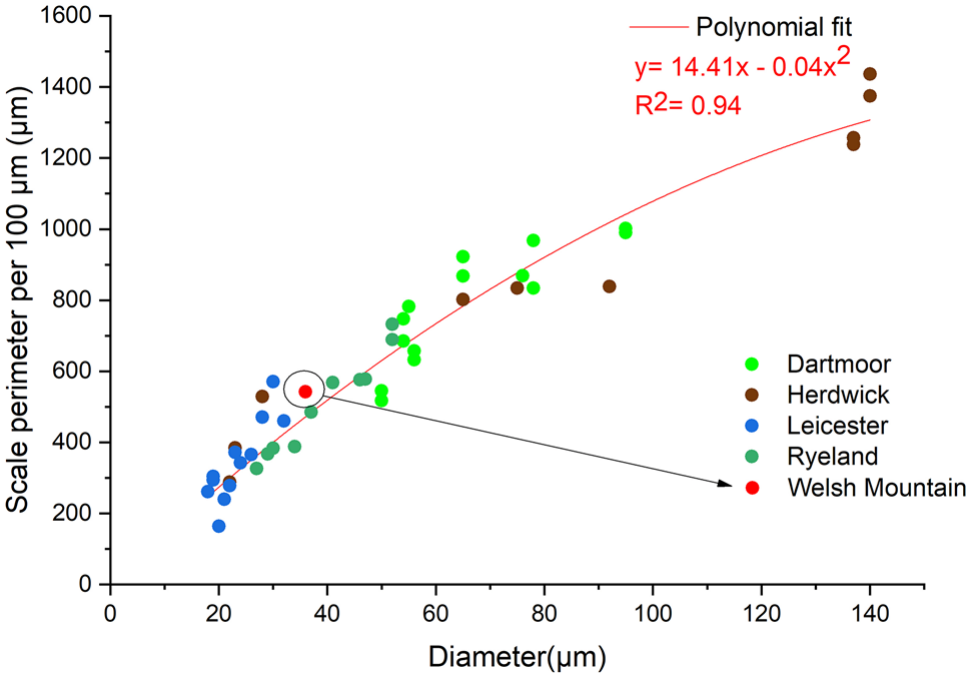
Relationship between total scale perimeter and fibre diameter.

**Figure 6:**
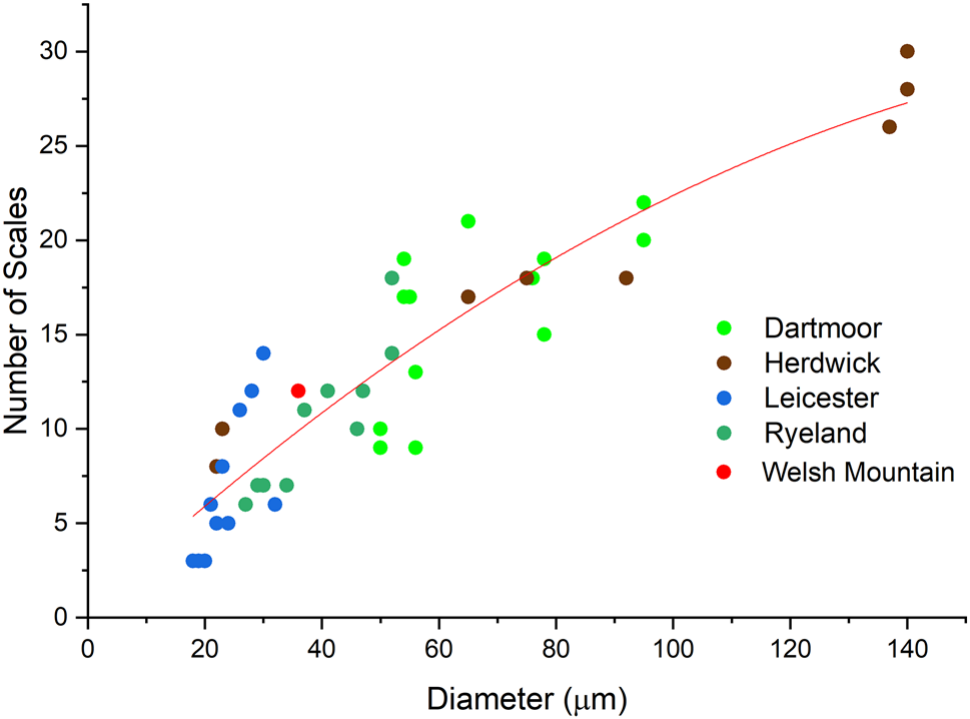
Relationship between number of scales per 100 µm length and fibre diameter.

**Figure 7:**
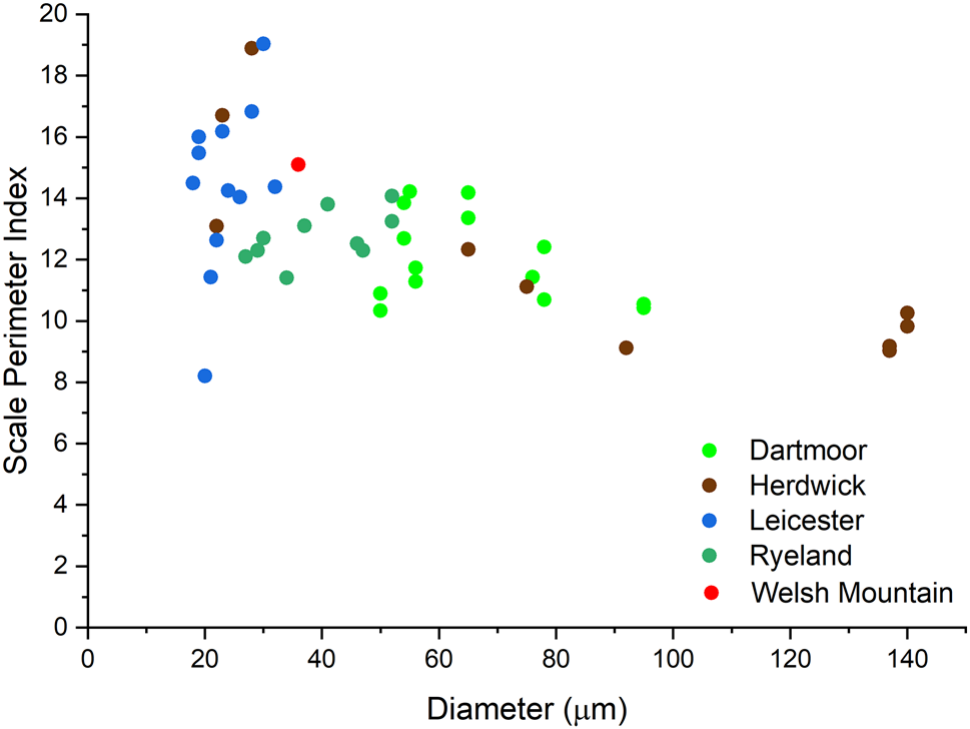
Relationship between scale perimeter index and fibre diameter.

The total scale perimeter per 100 µm length of fibre, SPI and number of scales were calculated for all the four wool types (Table S1 Supplementary material). Figures 5, 6 and 7 shows the relation between fibre diameter and total scale perimeter per 100 µm as well as SPI. All the data points of different breeds were plotted together to establish a correlation between these parameters.

The total scale perimeter per 100 µm and the number of scales showed a polynomial correlation (with R^2^ value of 0.94) with the fibre diameter, and the nature of the curve was hyperbolic (Figs. 5 and 6). From Fig. 5, it can be observed that the total scale perimeter increased with the increase in the fibre diameter and as the fibre diameter reached a high value, the rate of increase of total scale perimeter decreased making the curve similar to an asymptote. The trend line showed in Fig. 5 is represented by the equation:

4 $$y= 14.41 x-0.04{x}^{2}$$

where y is total scale perimeter per 100 µm and x is the measured diameter

This equation can be used as a framework to predict the total scale perimeter per 100 µm of any unknown type of wool fibre using its diameter value. This curve can also be used to know which type of scale pattern (predominantly) the wool fibre will exhibit depending on its diameter. Both the parameters can be used to characterise and understand the surface morphology of wool fibres.

As shown in Fig. 7, SPI decreased with an increase in the fibre diameter. Mainly the SPI values for fine fibres (19–29 µm diameter) ranged from 14 to 20, for medium fibres (30–65 µm diameter) from 11 to 15 and for coarser fibres (> 65 µm diameter) from 6 to 11. The correlation curves will be more accurate when more data from wool fibres of different breeds (besides the 4 fibre types used to develop the correlations) can be incorporated into it. To verify the established correlations, Welsh mountain wool fibre obtained from British Wool was studied under SEM to observe the scale pattern and measure the scale parameters. The total scale perimeter per 100 µm, SPI and the number of scales for this wool variety are 542 µm, 15.1 and 12 respectively.

The scale pattern observed for Welsh Mountain wool fibre was coronal-reticulate, which was expected as the measured diameter of the fibre was 36 µm between 25 and 60 µm (Fig. S3). SPI of Welsh Mountain wool fibre was 15 to 20, which was in the range for a medium diameter wool fibre. The total scale perimeter per 100 µm, SPI and number of scales value of Welsh mountain wool also fitted well with the data in the correlation curve, as shown in Figs. 5, 6 and 7. The total scale perimeter value calculated from Eq. (4) came to be 482 µm which is 60 µm less than the measured value 542 µm (Table S1). The deviation from the original fitted equation was attributed to the inherent variation of diameter within a wool fibre. Thus, more data set from different varieties of wool fibre could be used in the future research to make this correlation-based framework more accurate. Therefore, the results clearly indicated that all three studied scale parameters showed specific correlations with the diameter of wool fibre.

### Correlation between dyeing kinetics and scale parameters

**Figure 8:**
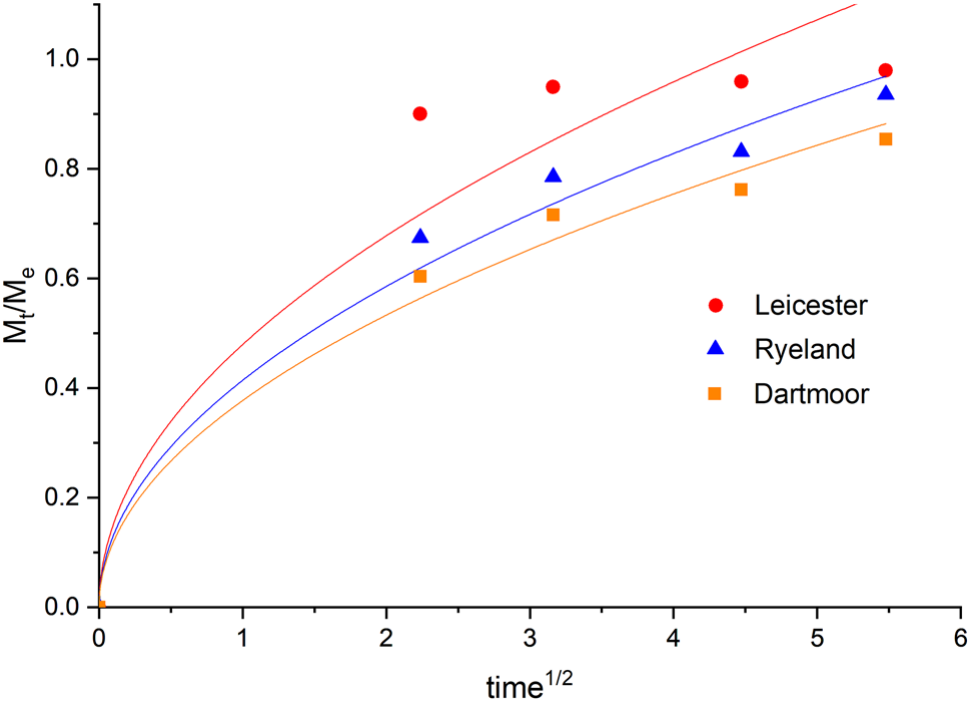
Relative dye uptake (M_t_/M_e_) vs time^1/2^ plots for Leicester, Ryeland and Dartmoor.

As Herdwick wool fibres showed a very wide diameter distribution, high CV% for mean fibre diameter (Table 2) and at the same time presence of kemp fibres, further experiments were done with the other three fibres. The diffusion coefficient of Acid Violet 90 on Leicester, Ryeland and Dartmoor were found to be 0.09, 0.13 and 0.59 × 10^–8^ (cm^2^ sec^–1^) (respectively) using Hill’s equation (Table 3). This modified method for a finite dye bath calculates the apparent diffusion coefficient which is assumed to remain constant in the early stage of dyeing which is the dye diffusion in the solution. The apparent diffusion coefficient for the fibres were obtained by fitting the Relative dye uptake (M_t_/M_e_) curve against time^1/2^ and using Eq. (2) as shown in Fig. 8.

**Table 3:** Calculated diffusion coefficient, average total scale perimeter per 100 µm and number of scales per 100 µm of Leicester, Ryeland and Dartmoor wool fibres.

Breeds*	Diffusion coefficient × 10^–8^ (cm^2^ sec^–1^)	Avg. total scale perimeter per 100 µm (µm)	Avg. number of scales per 100 µm
Leicester	0.091 ± 0.001	309 ± 27	6 ± 1.0
Ryeland	0.133 ± 0.001	510 ± 44	10 ± 1.1
Dartmoor	0.592 ± 0.011	754 ± 39	16 ± 1.2

**Figure 9:**
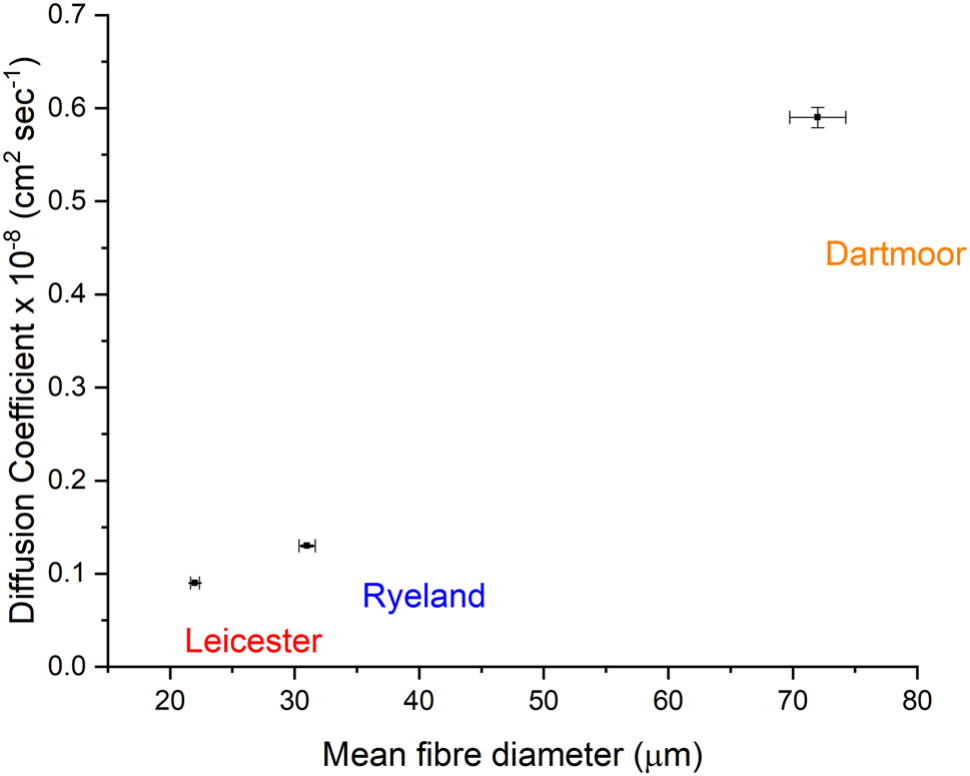
Relationship between apparent diffusion coefficient of Acid Violet 90 dye and mean fibre diameter (The error bars represent the standard error of the mean).

Researchers working with dyeing theories for cellulosic and manmade fibres have established that the factors influencing fibre dye uptake and rate of dyeing are fibre diameter and cross-sectional shape of the fibre^53, 54^. Finer fibres tend to absorb high amount of dye due to its higher surface area per unit volume, thus indicating a higher D for the dye molecules in finer diameter fibres^55, 56^. The current correlation curve plotted between diffusion coefficient and mean fibre diameter (Fig. 9) shows a linear increase in D with the increase in diameter of the fibres. The mean fibre diameter values were used during the calculation of D. This clearly suggests that for a wool acid dye system a surface morphology factor contributes towards changing the D of Acid Violet 90 dye.

**Figure 10:**
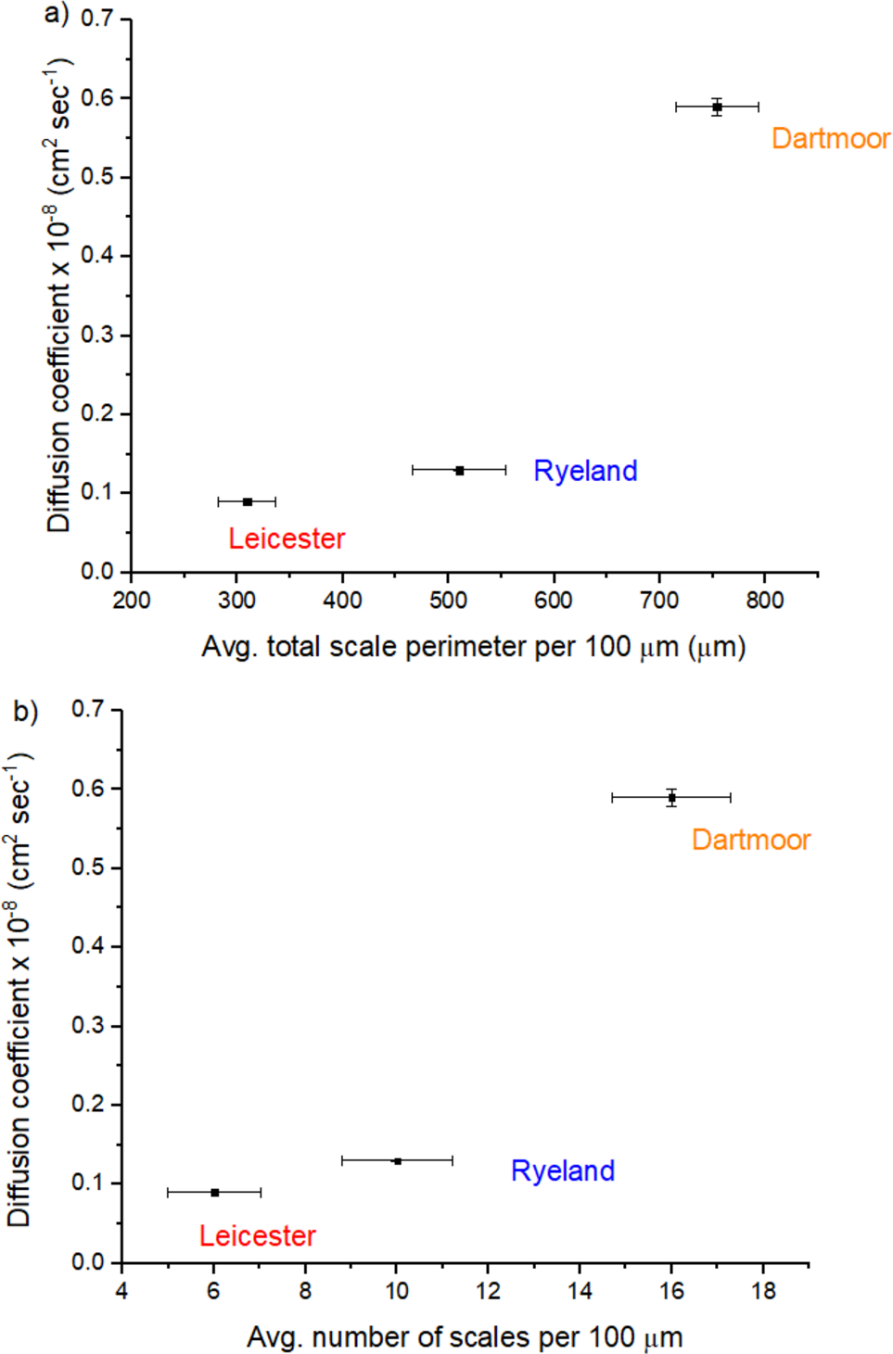
Relationship between apparent diffusion coefficient of Acid Violet 90 dye and (**a**) average total scale perimeter per 100 µm, (**b**) average number of scale per 100 µm (The error bars represent the standard error of the mean).

Further the D values of Acid Violet 90 were correlated to the average total scale perimeter per 100 µm and average number of scales per 100 µm, represented in Fig. 10a and b. An increase in D can be noticed with an increase in average total scale perimeter per 100 µm and average number of scales per 100 µm values. From Leicester to Ryeland to Dartmoor with an increase in diameter there was an increase in the number of scale per 100 µm associated with an increase in effective diffusion pathway. More the diffusion pathway available the higher will be the mobility of the acid dye molecules resulting in an increase in D value for the fibre. Similar observations have been reported by researchers where introduction of cracks on the surface of wool fibre resulted in an enhancement of the apparent diffusion coefficient value^57, 58^. These cracks were introduced by various physical and chemical surface modification techniques on wool fibre^35, 59^. In this study, the increase in effective diffusion pathway was caused by the natural variation in the scale patterns. Higher diffusion pathway in a wool fibre is associated with a reduction in hydrodynamic boundary layer as well as surface barrier effect thus allowing the dye molecules to reach the surface of the fibre at a faster speed^35, 42, 60^. Dartmoor having the highest diffusion pathway (total scale perimeter per 100 µm) shows highest diffusion coefficient followed by Ryeland and Leicester.

The rate of increase of D between Leicester and Ryeland was significantly less compared to the rate of increase between Leicester and Dartmoor. This can be supported with the findings made earlier that Ryeland showed the presence of both coronal and coronal reticulate scale pattern. Ryeland with coronal reticulate pattern have a higher avg. total scale perimeter per 100 µm than Leicester whereas presence of two different kind of scale pattern within the same wool fibre might be the possible reason for the rate of increase in D to be less.

It can be summarised from the earlier discussion that total scale perimeter per 100 µm have influenced the diffusion coefficient of Acid Violet 90 dye in the solution phase. This confirmation can have further impact on the overall rate of dyeing at the same time the amount of dye adsorbed on the fibre. Thus, further research can be performed in this area to correlate the scale parameters with dyeing thermodynamics and kinetics parameters.

### Discussion on the correlation of surface morphology with fibre functionality

Chemical properties of wool fibres (such as dyeing behaviour, moisture or chemical sorption, biodegradability, etc.) are significantly influenced by the effective diffusion pathway between the scales. The current study have opened up the possibility to investigate the effect of scale morphology on these essential properties of wool fibre^61^. Thus, the studied scale parameters could be useful to understand the functionalities and end use of different varieties of wool fibre.

Previous literature related to surface morphology of wool fibre mainly focused on characterising the scale height (Fig. 1) and only a few studies discussed how the scale height could influence fibre properties. For example, a permanganate salt pre-treatment was used to assess the effect of mean scale height on the fibre properties. This study reported that with increase in the concentration of the pre-treatment chemical the scale height decreased^44^. The effect of the permanganate salt was limited to the scales of the wool fibre, modifying the cuticle, and removing the hydrophobic scales. This change in scale structure resulted in a higher value of water absorption and an improvement in the surface roughness^44^. In the present research paper, new scale parameters related to the effective diffusion pathway of wool fibre (instead of scale height) have been characterised, leading to the possibility of better correlating scale morphology with important fibre properties and predicting various applications of different varieties of wool fibres:

#### Water sorption

Wool has a very high-water absorption property and can absorb more water as compared to any other natural fibre^62^. This is attributed firstly to the presence of hydrophilic groups in the protein structure of the fibre and secondly, to the surface structure of the wool fibre^63, 64^. When wool fibre absorbs water, the fibre swells and creates a path for the water molecules to diffuse through the intercellular gaps and breaks the hydrogen bonds in the amino acid structure. This in turn creates more interaction sites and increases the absorption of water molecules^65^. In both dyeing and water sorption phenomena, the surface morphology is likely to have a very high impact on the final uptake values. A fibre with more scale perimeter per 100 µm is expected to have a higher tendency to absorb water. This parameter, therefore, can be useful for the manufacturing of technical textile products such as medical textiles where high water or liquid sorption is a key requirement.

#### Biodegradation

The hydrophobic crosslinked epicuticle of wool fibre also resists biodegradation of wool fibre by the action of bacteria^66^. Thus, it is expected that more is the number of diffusion paths present on the surface of wool fibre more it will be susceptible to enzymatic hydrolysis or microbial attack, leading to a faster degradation rate^8, 67^. This will potentially allow wool products to be tailored with specific degradation properties.

Therefore, with further research and experimental verifications, the studied scale parameters can be used to classify different varieties of wool fibre according to their properties and end usage. This approach and knowledge will be very important for the wool industry in terms of using different breeds of wool fibre in diversified technical textile applications like filtration (air, blood, chemical, etc.), geotextiles, medical textiles, composites, garments and so on.

## Conclusion

In this research, wool fibres collected from four different British sheep breeds (Dartmoor, Leicester, Ryeland and Herdwick) were characterised using SEM to study their scale pattern and number of scales, and two novel scale parameters, namely total scale perimeter per 100 µm and SPI were calculated. The studied varieties of wool exhibited differences in their scale pattern, number of scales, scale perimeter and SPI. The patterns of scales visible on the surface were identified as coronal for Leicester and reticulate for Dartmoor wool, whereas Herdwick and Ryeland fibres showed more than one scale pattern due to their wider diameter distribution.

The change in scale pattern from coronal to reticulate was associated with the change in the fibre diameter and was attributed to the increase in number of scales within a specific fibre length. Total scale perimeter per 100 μm length showed a polynomial correlation with the fibre diameter. On the other hand, SPI values were observed to decrease with the increase in fibre diameter and showed specific ranges for finer, medium and coarser fibres.

The characterised scale parameters showed a good correlation with the apparent diffusion coefficient of Acid Violet 90 dye. The D values showed a steady increase with the increase in mean fibre diameter, avg. total scale perimeter per 100 µm and avg. number of scales per 100 µm. Wool fibre with highest avg. total scale perimeter per 100 μm (due to increased effective diffusion pathway) has shown the highest apparent diffusion coefficient (D) of Acid Violet 90. This clearly suggests that the effective diffusion pathway is an influencing factor during the early stage of dye diffusion.

This correlation between the fibre properties and the surface morphology can be utilised to design various technical textile products (medical textiles, filter fabrics, geotextiles, composites, etc.). This can also contribute towards developing a framework where wool fibres obtained from an unutilised or new breed can be studied and used in the application fields mentioned above. This research work, therefore, opens up further opportunities to investigate into the correlation of dyeing and other properties of wool fibre with the scale morphology.

### Supplementary Information

Supplementary Information.

## Data Availability

The supplementary materials contain the relevant experimental data whereas any other requirement of data are available upon reasonable request to the corresponding author.
