# Supplementary material for: Development of novel parameters for characterising scale morphology of wool fibre and its correlation with dye diffusion coefficient of acid dye
Source: Sci Rep. 2023 Oct 27;13:18444. doi: 10.1038/s41598-023-45689-w (PMC10611768; doi:10.1038/s41598-023-45689-w)
Supplement: Supplementary file 1 — Supplementary Information. [file 41598_2023_45689_MOESM1_ESM.docx]

**Development of Novel Parameters for Characterising Scale Morphology of Wool Fibre and its Correlation with Dye Diffusion Coefficient of Acid Dye**

Subhadeep Paul^1,^ *, Andrew Hewitt^1^, Sohel Rana^2^ and Parikshit Goswami^1^

^1^ Technical Textiles Research Centre, School of Arts and Humanities, University of Huddersfield, Queensgate, Huddersfield HD1 3DH, UK

^2^ Department of Textile & Fibre Engineering, Indian Institute of Technology Delhi, Hauz Khas, New Delhi - 110016

[A.Hewitt@hud.ac.uk](mailto:A.Hewitt@hud.ac.uk) , [sohelrana@iitd.ac.in](mailto:sohelrana@iitd.ac.ink) , [P.Goswami@hud.ac.uk](mailto:P.Goswami@hud.ac.uk)

* Corresponding author- [Subhadeep.Paul@hud.ac.uk](mailto:Subhadeep.Paul@hud.ac.uk)

**Supplementary material**

**Table S1**: Wool fibre scale and diameter measurements from SEM images

| **Fibre diameter**  **(µm)** | **Total scale perimeter (µm) per 100 µm length** | **Scale Perimeter Index (scale perimeter /fibre diameter)** | **No. of scales per 100 µm** | **Wool breed** |
| --- | --- | --- | --- | --- |
| 50 | 545 | 10.9 | 9 | Dartmoor |
| 54 | 685 | 12.7 | 17 | Dartmoor |
| 54 | 748 | 13.9 | 19 | Dartmoor |
| 55 | 782 | 14.2 | 17 | Dartmoor |
| 56 | 632 | 11.3 | 9 | Dartmoor |
| 56 | 657 | 11.7 | 13 | Dartmoor |
| 65 | 868 | 13.4 | 21 | Dartmoor |
| 65 | 922 | 14.2 | 17 | Dartmoor |
| 76 | 869 | 11.4 | 18 | Dartmoor |
| 78 | 834 | 10.7 | 15 | Dartmoor |
| 22 | 288 | 13.1 | 8 | Herdwick |
| 23 | 384 | 16.7 | 10 | Herdwick |
| 28 | 529 | 18.9 | 12 | Herdwick |
| 65 | 802 | 12.3 | 17 | Herdwick |
| 75 | 834 | 11.1 | 18 | Herdwick |
| 92 | 839 | 9.1 | 18 | Herdwick |
| 137 | 1238 | 9.0 | 26 | Herdwick |
| 137 | 1257 | 9.2 | 26 | Herdwick |
| 140 | 1375 | 9.8 | 28 | Herdwick |
| 140 | 1436 | 10.3 | 30 | Herdwick |
| 27 | 326 | 12.0 | 6 | Ryeland |
| 30 | 383 | 12.7 | 7 | Ryeland |
| 31 | 379 | 12.2 | 7 | Ryeland |
| 34 | 388 | 11.4 | 7 | Ryeland |
| 37 | 485 | 13.1 | 11 | Ryeland |
| 41 | 568 | 13.8 | 12 | Ryeland |
| 46 | 576 | 12.5 | 10 | Ryeland |
| 47 | 578 | 12.3 | 12 | Ryeland |
| 52 | 689 | 13.3 | 18 | Ryeland |
| 52 | 732 | 14.1 | 14 | Ryeland |
| 18 | 261 | 14.5 | 3 | Leicester |
| 19 | 294 | 15.5 | 3 | Leicester |
| 19 | 304 | 16 | 3 | Leicester |
| 20 | 164 | 8.2 | 3 | Leicester |
| 21 | 240 | 11.4 | 6 | Leicester |
| 22 | 278 | 12.6 | 5 | Leicester |
| 23 | 372 | 16.2 | 8 | Leicester |
| 24 | 342 | 14.3 | 5 | Leicester |
| 26 | 365 | 14 | 11 | Leicester |
| 28 | 471 | 16.8 | 12 | Leicester |
| 36 | 542 | 15.1 | 12 | Welsh Mountain |


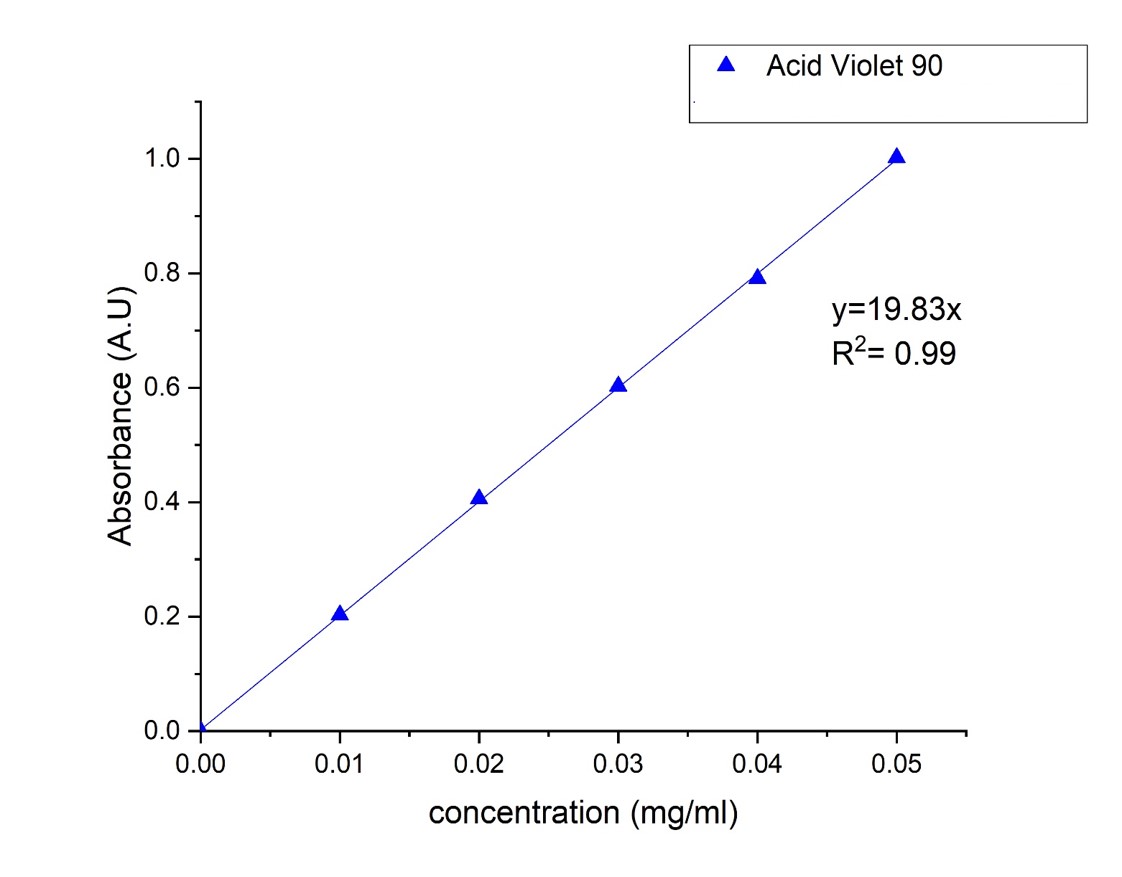


**Figure S2:** Calibration curve of Acid Violet 90 dye plotting absorbance vs concentration of the dye solution


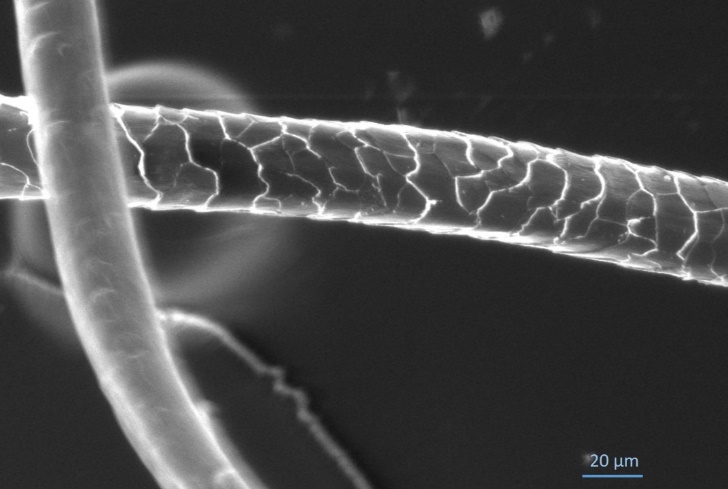


**Figure S3**: SEM image of Welsh Mountain wool fibre
